# The Linkage Between Inflammation and the Progression of Type 2 Diabetes Mellitus

**DOI:** 10.3390/cimb47100859

**Published:** 2025-10-17

**Authors:** Lucy Baldeón-Rojas, Valeria Alulema, Francisco Barrera-Guarderas, Diana Aguirre-Villacís, Cristina Cañadas-Herrera, Ricardo Bedón-Galarza, Francisco Pérez-Tasigchana, Jorge Pérez-Galarza

**Affiliations:** 1 Research Institute of Biomedicine, Central University of Ecuador, Quito 170201, Ecuador; vealu-lema@uce.edu.ec (V.A.); dfaguirre@uce.edu.ec (D.A.-V.); criss.mishell@gmail.com (C.C.-H.); jmperez@uce.edu.ec (J.P.-G.); 2 Faculty of Medicine, Central University of Ecuador, Quito 170403, Ecuador; rgbedon@uce.edu.ec; 3 Faculty of Medicine, Pontifical Catholic University of Ecuador, Quito 170143, Ecuador; fbfamapuce@gmail.com; 4 Internal Medicine Department, Hospital Julio Endara, Ministry of Health, Quito 170801, Ecuador; 5 Internal Medicine Department, Hospital General Docente de Calderón, Quito 170201, Ecuador; 6 Centro de Investigación en Salud Pública y Epidemiología Clínica, Facultad de Ciencias de la Salud Eugenio Espejo, Universidad UTE, Quito 170129, Ecuador; raul.perez@ute.edu.ec

**Keywords:** type 2 diabetes, inflammation, cytokines, microRNA

## Abstract

Type 2 diabetes mellitus (T2D) is a chronic metabolic disorder in which inflammation plays a central role in its onset, progression, and complications. Identifying reliable biomarkers is essential to improve risk prediction, disease monitoring, and early intervention. A total of 169 Ecuadorian participants were stratified into four clinical groups: non-diabetic controls (NDC), controlled T2D (C-T2D), uncontrolled T2D (NC-T2D), and diabetic kidney disease (DKD). Circulating levels of cytokines (IL-6, IL-8, TNF-α), adipokines (leptin, adiponectin), and PBMC-derived microRNAs (miR-146a, miR-155) were quantified. Associations with disease stage were evaluated using ROC curve analysis and logistic regression. Leptin showed the strongest association with T2D (OR = 13.76, 95% CI: 6.47–29.26), followed by IL-8 (OR = 6.73, 95% CI: 3.30–13.70) and IL-6 (OR = 4.43, 95% CI: 2.26–8.97). Adiponectin distinguished NC-T2D from DKD (OR = 4.15, 95% CI: 1.77–9.71), underscoring its potential as an indicator of renal complications. Interestingly, TNF-α levels declined across disease stages, possibly reflecting subclinical inflammation in Ecuadorian NDC with high rates of obesity and dyslipidemia. PBMC-derived miR-146a was upregulated in T2D patients, contrasting with prior serum-based studies and emphasizing the importance of compartment-specific analysis. miR-155 was elevated in C-T2D, suggesting a compensatory immune-regulatory mechanism that diminishes with poor glycemic control and advanced disease. Inflammatory cytokines, adipokines, and microRNAs act in distinct yet complementary ways in T2D. Leptin, IL-6, and IL-8 emerge as strong predictors of disease, while miR-146a and miR-155 provide additional insight into immune-inflammatory regulation. Integrated biomarker panels may enhance patient stratification and support personalized monitoring of T2D progression.

## 1. Introduction

Type 2 diabetes (T2D) affects more than 530 million individuals worldwide, with a global prevalence of approximately 10.5% [1]. Four out of every five people with diabetes live in low- and middle-income countries [2]. In Ecuador, T2D ranked as the second leading cause of mortality in 2021, affecting 4.7% of adults aged 20–79 years [3]. Individuals with T2D are at high risk of developing complications such as retinopathy, neuropathy, nephropathy, and cardiovascular disease, which substantially reduce quality of life and increase long-term healthcare demands [4]. Among these, diabetic kidney disease (DKD)—which affects about 40% of individuals with diabetes—is the most common cause of end-stage renal disease (ESRD) and a major determinant of mortality [5,6]. Although kidney biopsy remains the diagnostic gold standard, its invasive nature underscores the need for reliable, non-invasive biomarkers to monitor DKD progression [7,8].

Inflammation plays a central role in the pathogenesis of T2D and its complications [6,9]. Persistent hyperglycemia and the accumulation of advanced glycation end products (AGEs) promote cellular stress and the release of damage-associated molecular patterns (DAMPs). These molecules activate pattern-recognition receptors such as Toll-like receptors (TLRs), thereby triggering innate immune responses [10]. Activation of the TLR–NF-κB pathway enhances the production of pro-inflammatory cytokines, including interleukin (IL)-6, IL-1β, IL-18, and tumor necrosis factor-α (TNF-α), and stimulates transforming growth factor-β1 (TGF-β1), a key mediator of renal fibrosis [11,12,13]. Elevated serum IL-8 concentrations have been associated with disease severity and may serve as prognostic indicators in kidney disease [14,15].

Several microRNAs (miRNAs) have been identified as critical modulators of inflammation in metabolic disorders. In particular, miR-146a negatively regulates the production of cytokines such as TNF-α, IL-6, and IL-1β, thereby maintaining immune balance [16,17]. Conversely, miR-155 acts as a pro-inflammatory effector by inhibiting suppressor of cytokine signaling 1 (SOCS1), a pivotal negative regulator of the JAK/STAT and NF-κB pathways [18,19]. Dysregulation of these miRNAs has been reported in both tissues and systemic circulation of diabetic individuals, suggesting their potential as biomarkers of disease progression and targets for therapeutic modulation.

In parallel, alterations in adipokine profiles are commonly observed in diabetic nephropathy and may exacerbate inflammation, appetite dysregulation, and atherosclerosis [20]. Hyperleptinemia frequently appears early in T2D and worsens with disease duration [21]. Conversely, adiponectin—an anti-inflammatory adipokine that enhances insulin sensitivity—is often reduced in chronic kidney disease (CKD) [22]. Thus, the leptin-to-adiponectin ratio has been proposed as a surrogate indicator of adipose tissue dysfunction and renal inflammation [20].

Given these observations, the present study aimed to evaluate inflammatory cytokines (TNF-α, IL-6, IL-8), adipokines (adiponectin, leptin), and microRNAs (miR-155, miR-146a) as candidate biomarkers for disease stage and progression in T2D.

## 2. Materials and Methods

### 2.1. Patients

A cross-sectional observational study was conducted, involving 598 fasting patients aged 40 years or older who were diagnosed with type 2 diabetes mellitus (T2DM). The diagnosis of T2DM was performed according to the criteria established by the Committee of Experts for the identification and categorization of the disease, at a type C public health center (Chimbacalle) located in Quito, Ecuador [23]. From this group, 169 patients were selected based on the inclusion criteria and divided into four categories: (1) controlled diabetic patients (T2DM-C), defined as those with an HbA1c value ≤ 6%; (2) uncontrolled diabetic patients (T2DM-NC), with HbA1c values > 7% but <8% and without kidney disease (defined as a glomerular filtration rate [GFR] > 60 mL/min/1.73 m^2^); (3) diabetic patients with kidney disease (DKD), characterized by an HbA1c value > 7% and a GFR < 60 mL/min/1.73 m^2^; and (4) non-diabetic controls (NDC), consisting of non-diabetic patients without kidney disease and with fasting glucose < 100 mg/dL for biomarker evaluation. In addition, patients with high blood pressure were included as a non-excluding condition in this study.

### 2.2. Biochemical Parameters and Cytokines

All participants fasted before blood sample collection. Peripheral venous blood (10 mL) was collected into tubes without anticoagulant and transported to the laboratory under cold chain conditions (4 °C). Samples were processed within two hours post-collection. Serum was aliquoted and stored at −20 °C until analysis. Biochemical parameters—including glucose, HbA1c, lipid profile (total cholesterol, triglycerides, HDL, LDL), SGOT, SGPT, urea, and creatinine—were quantified using validated protocols of the Biomedical Research Institute, Central University of Ecuador. IL-6, IL-8, TNF-α, leptin, and adiponectin were determined by ELISA, using the commercial R&D system (Minneapolis, MN, USA), following the manufacturer’s instructions.

### 2.3. MicroRNA RT qPCR Assays

Peripheral venous blood (4 mL) was collected in heparin tubes from fasting patients. Peripheral blood mononuclear cells (PBMCs) were isolated from low-density gradient centrifugation using Ficoll [24]. Total RNA was isolated from PBMCs using the Mirvana kit Ambion (a Thermo Fisher Scientific brand) (Waltham, MA, USA). The endogenous gene RNU-44 (cat no. 4427975, assay ID 1094) was used to normalize the cycle threshold (CT) values of RNA. Primers hsa-miR-146a (cat no. 4427975, assay ID 000468) and hsa-miR-155 (cat no. 4427975, assay ID 002623) for stem-looped reverse transcription were used to obtain cDNA for mature microRNAs. RNA was transcribed using the TaqMan microRNA reverse transcription kit (Applied Biosystems, Thermo Fisher Scientific Inc.) (Waltham, MA, USA). qPCR was performed using predesigned TaqMan microRNA assays and the TaqMan Universal Master Mix, No AmpEraseUNG. The qPCR was performed under the following conditions: 2 min at 50 °C, 10 min at 95 °C, followed by 40 cycles of 15 s at 95 °C, and finally 1 min at 60 °C in the Quant Studio 5 Real-Time PCR System (Applied Biosystems), (Waltham, MA, USA). Outliers were identified and removed using the interquartile range (IQR) method. For normalization, the cycle threshold (Ct) value of the endogenous control gene (RNU44) was subtracted from the Ct value of the target microRNA in each sample, generating ΔCt (dCt) values. The mean ΔCt of the control group was then used as the reference to compute ΔΔCt (ddCt) values, from which relative expression (fold change) was derived using the 2^−ΔΔCt method.

### 2.4. Statistical Analysis

The Kolmogorov–Smirnov test was used to determine the normality of the variables. The demographic, clinical, and molecular variables of the four groups were compared using ANOVA and Kruskal–Wallis with the Bonferroni post hoc test. We conducted ROC curves to identify the cut-off points that enable the identification of microRNAs and interleukins as potential biomarkers; logistic regression analysis was used to determine the predictive factors in the studied groups. Bivariate models were used for binary variables in the logistic regression analysis, whereas multivariate models were used for variables with more than two categories, controlling for age, sex, BMI, smoking status, and exercise. Statistical significance was defined as values of *p* < 0.005. IBM’s SPSS v25 software was used for statistical analysis. GraphPad Prism software (version 5.02) was used to create figures.

### 2.5. Ethical Declaration

All patients who were recruited at the type C Public Health Center in Quito, Ecuador (Chimbacalle) signed an informed consent form that was previously authorized by the Human Research Ethics Committee of Central University of Ecuador under approval number 0007-FCM-DD-2018.

## 3. Results

A total of 598 individuals were initially screened for participation. After applying inclusion and exclusion criteria, 169 participants were included for analysis of circulating cytokines, adipokines, and microRNAs. Participants were stratified into four clinical groups according to disease status: non-diabetic controls (NDC), patients with controlled type 2 diabetes (C-T2D), patients with uncontrolled type 2 diabetes (NC-T2D), and individuals with diabetic kidney disease (DKD) characterized by glomerular injury.

Demographic analysis showed no significant differences in sex distribution among the groups. However, participants with DKD were significantly older than all other groups (*p* < 0.001). Clinical and biochemical parameters followed expected patterns of disease progression. Fasting glucose and glycated hemoglobin (HbA1c) levels were markedly higher in NC-T2D and DKD groups compared with NDC and C-T2D (*p* < 0.001), reflecting poor glycemic control. Interestingly, body mass index (BMI) was highest in C-T2D and declined in NC-T2D and DKD groups (*p* < 0.05), possibly indicating metabolic deterioration or disease-related catabolism. Diastolic blood pressure was significantly elevated in DKD compared to NDC (*p* = 0.036). Indicators of renal function, including serum creatinine, urea, and estimated glomerular filtration rate (MDRD equation), were significantly altered in the DKD group (*p* < 0.001), confirming the presence of kidney impairment (Table 1).

### 3.1. Inflammatory Cytokines and Adipokines

Circulating cytokine and adipokine levels varied according to disease severity. IL-6, IL-8, leptin, and adiponectin concentrations increased progressively from NDC to C-T2D, NC-T2D, and DKD (IL-6, *p* ≤ 0.001; IL-8, *p* < 0.001; leptin, *p* < 0.001; adiponectin, *p* ≤ 0.006). In contrast, TNF-α concentrations declined significantly as disease severity advanced (C-T2D, *p* = 0.011; NC-T2D, *p* = 0.011; DKD, *p* = 0.002), suggesting a complex inflammatory modulation across the diabetic spectrum (Table 2).

### 3.2. MicroRNA Expression Profiles

**Figure 1:**
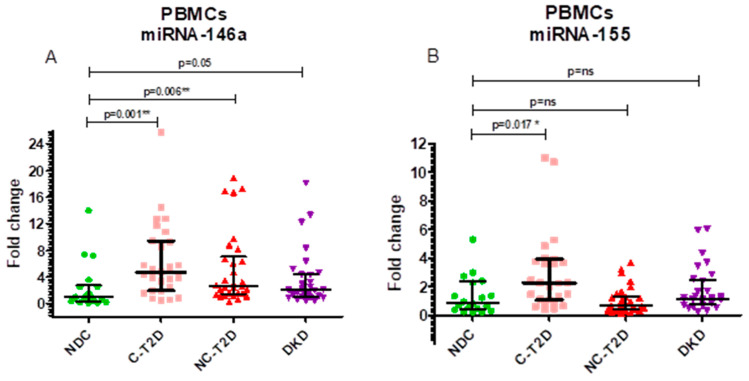
Fold-change values with interquartile ranges (IQR). (**A**). miR-146a, normalized to the endogenous reference gene RNU-44, in PBMCs. (**B**). miR-155, normalized to the endogenous reference gene RNU-44, in PBMCs. Group comparisons were performed using the Mann–Whitney U test against the NDC group. Study groups include non-diabetic controls (NDC), controlled T2D (C-T2D), uncontrolled T2D (NC-T2D), and T2D with diabetic kidney disease (DKD). Statistical significance: *p* < 0.05 (*), *p* < 0.01 (**); ns = not significant.

The relative expression of miR-146a (fold change, 2^−ΔΔCt) differed significantly between groups (Kruskal–Wallis, *p* = 0.0033). Median ± IQR values were 1.04 ± 2.45 in NDC, 4.75 ± 7.51 in C-T2D, 2.68 ± 5.65 in NC-T2D, and 2.07 ± 3.43 in DKD. Post hoc comparisons revealed significant upregulation in C-T2D (*p* = 0.0016) and NC-T2D (*p* = 0.0068) versus NDC, while DKD exhibited a borderline increase (*p* = 0.0502). These findings indicate enhanced miR-146a expression during the early and intermediate stages of T2D, with attenuation in advanced renal disease (Figure 1A).

Similarly, miR-155 expression varied across groups (Kruskal–Wallis, *p* = 0.007). Median ± IQR values were 0.89 ± 1.99 (NDC), 2.28 ± 2.87 (C-T2D), 0.65 ± 0.94 (NC-T2D), and 1.15 ± 1.66 (DKD). Pairwise analyses showed significant upregulation in C-T2D compared to NDC (*p* = 0.0174), while no significant differences were found between NDC and NC-T2D or DKD (*p* > 0.25) (Figure 1B).

### 3.3. Diagnostic Performance of Biomarkers

**Figure 2:**
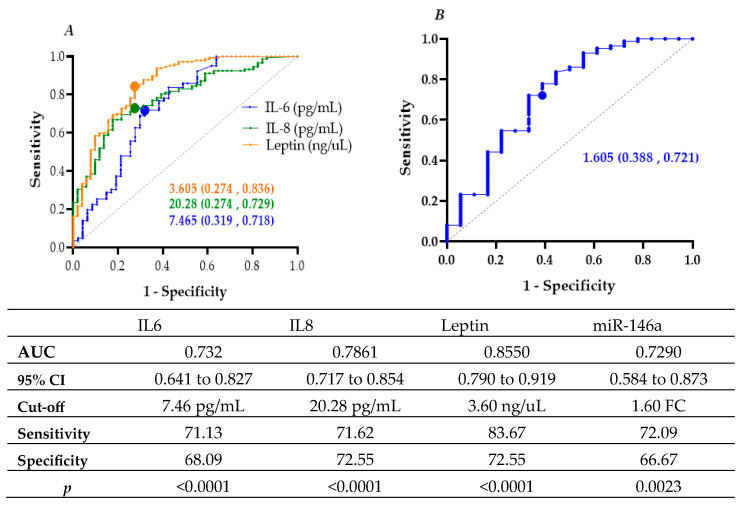
ROC curves with AUC and optimal cut-off points for (**A**) IL-6, IL-8, and leptin, and (**B**) miR-146a (fold change) associated with type 2 diabetes (T2D).

Receiver operating characteristic (ROC) curve analysis assessed the discriminatory capacity of circulating biomarkers for distinguishing T2D from non-diabetic controls. Leptin demonstrated the strongest performance (AUC = 0.8550; 95% CI: 0.7907–0.9193; *p* < 0.0001) at a cut-off of 3.605 ng/µL, yielding 83.7% sensitivity and 72.6% specificity. IL-8 (AUC = 0.7861; 95% CI: 0.7179–0.8543; *p* < 0.0001) and IL-6 (AUC = 0.732; 95% CI: 0.6411–0.8271; *p* < 0.0001) also exhibited good discriminatory capacity. Among microRNAs, miR-146a achieved an AUC of 0.7290 (95% CI: 0.5846–0.8734; *p* = 0.0023) with a 1.6-fold-change threshold, providing 72.1% sensitivity and 66.7% specificity (Figure 2).

Binary logistic regression further supported these associations. Elevated IL-8 (≥20.28 pg/mL) increased the odds of T2D 6.7-fold (OR = 6.733; 95% CI: 3.307–13.709; *p* < 0.001), while IL-6 (≥7.465 pg/mL) and leptin (≥3.605 ng/mL) were also significant predictors (OR = 4.436 and 13.765, respectively; *p* < 0.001). Similarly, miR-146a (≥1.605-fold change) conferred a 4.9-fold increase in T2D odds (OR = 4.857; 95% CI: 1.714–13.767; *p* = 0.003) (Table 3).

### 3.4. Biomarkers Associated with Diabetic Kidney Disease

**Figure 3:**
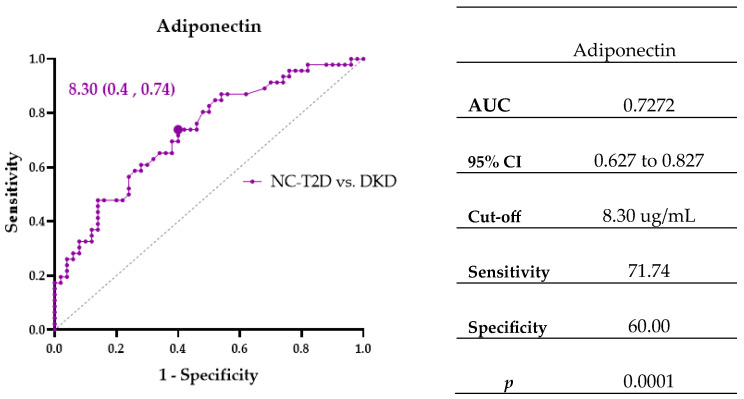
ROC curve showing the area under the curve (AUC) and cut-off points for biomarker Adiponectin associated with diabetes and kidney disease.

To identify biomarkers linked to diabetic kidney injury, ROC and logistic regression analyses were performed comparing NC-T2D and DKD groups. Adiponectin emerged as a significant discriminator, with concentrations ≥ 8.30 µg/mL conferring 4.15-fold higher odds of DKD (OR = 4.154; 95% CI: 1.776–9.718; *p* = 0.001) (Figure 3, Table 4).

## 4. Discussion

One of the primary triggers of T2D onset, progression, and complications is inflammation, and our research supports earlier findings that the disease’s primary hallmark is dysregulated cytokine activity. Our finding that TNF-α declined with T2D progression is unexpected, as this cytokine is typically considered a central driver of insulin resistance and metabolic inflammation. Prior studies, such as those by Eswar et al., have consistently reported elevated TNF-α in individuals with metabolic syndrome and T2D [25]. In contrast, our cohort did not exhibit this pattern, suggesting that population context may strongly influence inflammatory profiles. A notable explanation lies in the characteristics of our control group. Far from representing a metabolically “healthy” baseline, many participants displayed dyslipidemia (74% with high cholesterol, 90% elevated LDL, and 34% with hypertriglyceridemia) and excess body weight (BMI > 25 in most cases). In Ecuador, obesity and overweight are highly prevalent (22.3% and 39.5%, respectively) [26]. Obesity is now widely recognized as a chronic inflammatory state, with adipose tissue and free fatty acids fueling the production of TNF-α and related cytokines [27,28]. Thus, elevated TNF-α in our “normal” group may already exhibit subclinical inflammation, diminishing the relative differences detectable across the T2D spectrum.

IL-8 is synthesized and secreted by endothelial and epithelial cells, macrophages, and fibroblasts. Its pro-inflammatory action includes the recruitment and activation of neutrophils, T lymphocytes, and basophils, with the consequent cell apoptosis, fibrosis, and defective angiogenesis [29]. Farhan Mohammed et al. showed higher serum levels of IL-8 in T2D patients compared to healthy controls [29]. Another cross-sectional study showed increasing amounts of IL-8 as the patients’ renal function declined [14]. A study conducted by Loretelli et al. shows that targeting the IL-8 and C-X-C chemokine receptor types 1 and 2 (CXCR1/2) axis may reduce the burden of diabetic kidney disease [30]. These findings are consistent with our findings that individuals with uncontrolled diabetes and kidney disease had considerably higher levels of IL-8 with an OR = 6.733 (95% CI 3.307–13.709), indicating that chronic inflammation plays a crucial role in the development and advancement of microvascular damage in diabetic patients.

Importantly, several studies indicate that worsening of glucose control is positively and linearly associated with high levels of IL-6 [31]. Current evidence indicates a crucial role of IL-6 in podocyte injury [32]. Podocyte hypertrophy observed in diabetic nephropathy (DN) may be related to IL-6 signaling through the activation of Janus kinase 2/signal transducer and activator of transcription 3 signaling pathway (JAK2/STAT3) [33]. Interestingly, treatment with IL-6 antibody reduced apoptosis of these cells [34]. Our work supports previous researchers’ findings, which indicate that the length and severity of the diabetes condition are correlated with a linear upward expression of IL-6.

Many studies have been carried out regarding the relationship between leptin and diabetic complications. For example, a risk factor for the decrease in renal function has been identified to be the serum leptin levels [35]. On the other hand, some studies have demonstrated that there was no variation in leptin levels between diabetic individuals with and without nephropathy [36]. In our research, leptin was the biomarker that showed the strongest association, OR = 13.765 (95% CI 6.474–29.267), with T2D progression. Leptin is therefore an attractive option as a biomarker for the onset of diabetic complications.

Diabetic nephropathy (DN) is one of the long-term consequences of inadequate glycemic management in individuals with T2D [5]. Multiple studies have demonstrated a link between prolonged immune system activation and higher susceptibility to renal disease development. Adiponectin has anti-inflammatory, anti-apoptotic, and anti-fibrotic effects on the kidneys, reducing oxidative stress [37]. Jia et al. showed that CKD patients had higher circulating levels of adiponectin, which was associated with a higher risk of death [38]. As a potential defense against damage driven by inflammation and chronic oxidative stress, we also discovered in our study that the group of diabetic patients with kidney disease had much greater levels of adiponectin than the non-diabetic controls.

MicroRNAs regulate gene transcription and diverse biological functions, including inflammation, apoptosis, and proliferation [39]. In type 2 diabetes (T2D), miR-146a acts as a molecular switch of insulin signaling and inflammation, and its dysregulation promotes insulin resistance and complications such as nephropathy, neuropathy, cardiovascular disease, and retinopathy [40,41,42]. Experimental models show worsened renal injury in miR-146a−/− mice [43]. Mechanistically, miR-146a targets TRAF6, IRAK1, and IRAK2, modulating NF-κB signaling and dampening inflammation [44,45]. It also may impair insulin signaling via IRS-1 downregulation [46]. This study demonstrates that individuals with type 2 diabetes (T2D) exhibit increased expression of miR-146a in peripheral blood mononuclear cells (PBMCs), linking this microRNA to the inflammatory and metabolic processes that shape the disease. Compared with non-diabetic controls, both controlled (C-T2D) and non-controlled (NC-T2D) diabetic groups showed higher levels of miR-146a, while the diabetic kidney disease (DKD) group displayed only a borderline increase, possibly reflecting the heterogeneity of advanced disease stages where multiple pathophysiological mechanisms interact.

These findings stand in contrast with earlier studies reporting downregulation of circulating miR-146a in serum, underscoring the importance of biological context [40]. PBMC-derived miRNAs reflect intracellular immune-inflammatory activity and may capture closer links to metabolic dysregulation, while serum miRNAs are shaped by extracellular release and systemic circulation. This means that biomarkers measured in different biological compartments, such as blood serum versus immune cells, each provide unique but non-substitutable information about disease processes [47]. Functionally, miR-146a acts as an inflammatory regulator involved in insulin resistance and chronic low-grade inflammation [48]. Its upregulation in C-T2D may represent a compensatory mechanism to counteract immune activation, whereas variability in DKD could arise from altered renal processing or clearance of microRNAs. Thus, miR-146a emerges as a potential biomarker of metabolic control and progression in T2D, though confirmation in larger and longitudinal cohorts remains essential. Similarly, miR-155 expression was elevated in C-T2D but not in NC-T2D or DKD, suggesting a role in metabolic regulation that diminishes in uncontrolled diabetes or advanced complications. This points toward a possible immune-compensatory mechanism linked to glycemic stability.

The statistical findings obtained in this study reinforce the biological significance of the analyzed biomarkers. The ROC analysis revealed that leptin, IL-8, and IL-6 exhibited strong discriminatory capacity for distinguishing individuals with T2D from non-diabetic controls, with AUC values exceeding 0.73. These results highlight the clinical potential of inflammatory and adipokine markers as complementary diagnostic tools in metabolic disease. Likewise, miR-146a showed a moderate but meaningful discriminatory performance (AUC = 0.729), suggesting that circulating microRNAs may capture early immunometabolic alterations not reflected by conventional biochemical indices. The fold-change expression patterns of miR-146a and miR-155 further support this interpretation: miR-146a upregulation was most evident in controlled diabetes, reflecting a compensatory anti-inflammatory response, while its attenuation in DKD may indicate loss of regulatory capacity with chronic inflammation. Conversely, the transient rise in miR-155 in controlled disease and its subsequent decline in advanced stages could mirror the dynamic modulation of pro-inflammatory signaling. Together, these integrated statistical and biological findings suggest that a panel combining cytokines, adipokines, and microRNAs may provide enhanced sensitivity for monitoring disease progression and identifying early renal involvement in T2D.

Our results also highlight the predictive power of classical inflammatory markers. Leptin showed the highest accuracy for identifying T2D, consistent with its dual role in adiposity and inflammatory activation of adipose tissue. Elevated IL-6 and IL-8 further reinforced the contribution of chronic inflammation, with IL-6 impairing insulin signaling and IL-8 promoting vascular inflammation. While miR-146a showed only moderate predictive value, its integration with cytokine profiles may improve disease classification. Notably, adiponectin distinguished NC-T2D from DKD, indicating that its dysregulation may reflect progression toward renal complications. This highlights its value not only as a marker of metabolic imbalance but also as a potential predictor of vascular and renal risk [49].

Taken together, these findings emphasize that no single biomarker fully explains T2D pathophysiology. Instead, combining cytokines, adipokines, and microRNAs offers a more comprehensive picture of metabolic–inflammatory interactions. Future studies in larger and more diverse cohorts are needed to validate these results and assess the translational potential of multi-marker panels for early detection, patient stratification, and monitoring of disease progression.

## 5. Conclusions

This study reinforces the central role of inflammation in type 2 diabetes, revealing that cytokines, adipokines, and microRNAs act in complementary ways during disease progression. Leptin emerged as the strongest predictor of T2D, while IL-6 and IL-8 reflected persistent inflammation. Adiponectin distinguished advanced kidney disease, suggesting a protective yet complex role. PBMC-derived miR-146a was upregulated in T2D, contrasting with serum-based studies and emphasizing the influence of biological compartment on biomarker interpretation. Although the cross-sectional design, modest control group, and possible medication effects limit causal inference, the findings remain robust and hypothesis-generating. Overall, multimarker panels integrating cytokines, adipokines, and microRNAs may improve risk prediction, patient stratification, and personalized monitoring in T2D.

## Figures and Tables

**Table 1:** Clinical characteristics of study groups: Non-diabetic Controls (NDC), Controlled-diabetics (C-T2D), non-controlled diabetics (NC-T2D), and Diabetics with glomerular alteration (DKD) groups.

		NDC (n = 49)	C-T2D (n = 50)	NC-T2D (n = 50)	DKD (n = 49)	*p*-Value
Female (%)		39(79.52)	40 (80.0)	45 (90.0)	45(91.84)	0.175
Male (%)		10(20.41)	10(20.0)	5(10.0)	4(8.16)	
Age (years)		59 ± 8	60 ± 8	59 ± 10	72 ± 11	0.000 NDC vs. DKD *p* = 0.000 C-T2D vs. DKD *p* = 0.000 NC-T2D vs. DKD *p* = 0.000
Exercise	Yes	6 (12.24)	41 (83.67)	38 (79.17)	39 (79.59)	0.649
	Not	43 (87.76)	8 (16.33)	10 (20.83)	10 (20.41)	
Smoker	Yes	7 (14.29)	4 (8.16)	6 (12.24)	5 (10.20)	0.795
	Not	42 (85.71)	45 (91.84)	43 (87.76)	44 (89.80)	
Length of illness (years)			8 ± 5	9 ± 7	16 ± 11	0.000 C-T2D vs. NC-T2D *p* = 0.042 C-T2D vs. DKD *p* = 0.000 NC-T2D vs. DKD *p* = 0.000
Age at diagnosis (years)			53 ± 10	54 ± 12	52 ± 14	0.005 NC-T2D vs. C-T2D *p* = 0.018 NC-T2D vs. DKD *p* = 0.012 C-T2D vs. DKD *p* = 1.00
Medication	Metformin		39 (79.59)	17 (36.17)	3 (6.12)	0.000 C-T2D vs. Metf *p* = 0.000 DKD vs. Metf *p* = 0.000
	Metformin/Gilbenclamide		3 (6.12)	12 (25.53)	15 (30.61)	
	Insulin		0 (0)	1 (2.13)	6 (12.24)	
	Insulin/Metformine		7 (14.29)	16 (34.04)	23 (46.95)	
	Gilbenclamide/Insulin/Metformine		0 (0)	1 (2.13)	2 (4.08)	
Family history	Yes	39 (79.59)	32 (65.31)	29 (58)	26 (54.17)	0.006
	Not	10 (20.41)	17 (34.69)	21 (42)	22 (45.83)	
BMI (kg/m^2^)		31.1 ± 4.2	30.8 ± 5.6	30.4 ± 4.4	27.2 ± 4.2	0.001 NDC vs. DKD *p* = 0.007 C-T2D vs. DKD *p* = 0.002 NC-T2D vs. DKD *p* = 0.029
SBP (mmHg)		121 ± 12	121 ± 10	121 ± 12	124 ± 16	0.427
DBP (mmHg)		74 ± 6	74 ± 7	73 ± 74.74	70 ± 8	0.03 NDC vs. DKD *p* = 0.036
Glucose (mg/dL)		82 ± 9	100 ± 15	141 ± 84.84	161 ± 46	0.000 NDC vs. DKD *p* = 0.000 C-T2D vs. DKD *p* = 0.000 NC-T2D vs. DKD *p* = 0.000
HbA1C (%)		5.6 ± 0.5	5.8 ± 0.4	7.5 ± 0.3	8.8 ± 0.9	0.000 NDC vs. DKD *p* = 0.000 C-T2D vs. DKD *p* = 0.000 NC-T2D vs. DKD *p* = 0.000
Cholesterol (mg/dL)		193 ± 37	185 ± 38	174 ± 37.37	200 ± 37	0.136
Triglycerides (mg/dL)		174 ± 68	161 ± 85	168 ± 68.68	171 ± 76	0.801
HDL (mg/dL)		47 ± 13	48 ± 10	45 ± 8	48 ± 13	0.653
LDL (mg/dL)		106 ± 33	104 ± 35	92 ± 33	105 ± 31	0.369
SGOT (U/L)		21.90 ± 12.96	22.35 ± 13.93	23.25 ± 10.23	21.00 ± 24.08	0.583
SGPT (U/L)		12.85 ± 10.73	13.40 ± 7.61	15.20 ± 6.24	11.00 ± 7.21	0.657
Urea (mg/dL)		33.00 ± 8.90	32.00 ± 10.16	27.50 ± 9.27	40.00 ± 45.01	0.000 NDC vs. NC-T2D *p*= 0.020 NC-T2D vs. DKD *p* = 0.000 C-T2D vs. DKD *p* = 0.002
Creatine (mg/dL)		0.90 ± 0.15	0.90 ± 0.15	0.85 ± 0.0	1.10 ± 1.02	0.000 NDC vs. DKD *p* = 0.000 NC-T2D vs. DKD *p* = 0.000 C-T2D vs. DKD *p* = 0.002
MDRD (mL/min)		70.10 ± 11.04	73.35 ± 11.48	76.45 ± 10.75	52.80 ± 17.56	0.000 NDC vs. DKD *p* = 0.000 C-T2D vs. DKD *p* = 0.000 NC-T2D vs. DKD *p* = 0.000

T2D: type 2 diabetes, BMI: body mass index, SBP: systolic blood pressure, DBP: diastolic blood pressure, HbA1C: glycosylated hemoglobin, HDL: high-density lipoproteins, LDL: low-density lipoproteins, SGOT: Serum Glutamic Oxaloacetic Transaminase, SGPT: Serum Glutamic Pyruvic Transaminase, MDRD: Glomerular filtration rate. The mean +/− SD is displayed for the variables with a normal distribution, such as age, BMI, cholesterol, and LDL, whereas the median +/− IQR is displayed for the variables with a non-normal distribution.

**Table 2:** Inflammatory biomarkers of Non-diabetic Controls (NDC), controlled diabetics (C-T2D), non-controlled diabetics (NC-T2D), and diabetics with glomerular alteration (DKD).

Variable	NDC (n = 20)	C-T2D (n = 50)	NC-T2D (n = 50)	DKD (n = 49)	*p*-Value
IL-8 (pg/mL)	14.91 ± 12.05	30.66 ± 27.45	30.38 ± 27.94	30.66 ± 24.78	0.000 NDC vs. C-T2D *p* = 0.000 NDC vs. NC-T2D *p* = 0.000 NDC vs. DKD *p* = 0.000
IL-6 (pg/mL)	3.09 ± 10.63	11.62 ± 9.10	10.48± 14.13	12.16 ± 13.29	0.000 NDC vs. C-T2D *p* = 0.001 NDC vs. NC-T2D *p* = 0.001 NDC vs. DKD *p* = 0.000
TNF-α (pg/mL)	9.28 ± 3.17	6.73 ± 3.43	6.90 ± 2.70	5.58 ± 3.94	0.001 NDC vs. C-T2D *p* = 0.011 NDC vs. NC-T2D *p* = 0.011 NDC vs. DKD *p* = 0.002
Leptin (ng/mL)	1.32 ± 4.46	11.14 ± 6.96	9.84 ± 7.85	8.65 ± 8.12	0.000 NDC vs. C-T2D *p* = 0.000 NDC vs. NC-T2D *p* = 0.000 NDC vs. DKD *p* = 0.000
Adiponectin (ug/mL)	6.17 ± 5.54	9.30 ± 5.43	6.65 ± 4.12	11.30 ± 6.55	0.000 NDC vs. C-T2D *p* = 0.006 NDC vs. NC-T2D *p* = 0.000 NDC vs. DKD *p* = 0.001
miR-146a (fold change)	1.04 ± 2.45	4.75 ± 7.51	2.68 ± 5.65	2.07 ± 3.43	0.003 NDC vs. C-T2D *p* = 0.001 NDC vs. NC-T2D *p* = 0.006 NDC vs. DKD *p* = 0.050
miR-155 (fold change)	0.89 ± 1.99	2.28 ± 2.87	0.65 ± 0.94	1.15 ± 1.66	0.000 NDC vs. C-T2D *p* = 0.017 NDC vs. NC-T2D *p* = 0.422 NDC vs. DKD *p* = 0.263

Tumor necrosis factor alpha (TNF-α), interleukin-8 (IL-8), interleukin-6 (IL-6), microRNA-146a (miR-146a), and microRNA-155 (miR-155). All variables exhibited a non-normal distribution and are therefore expressed as median with interquartile range (IQR). Fold change values were calculated using the 2^−ΔΔCT method.

**Table 3:** Binary logistic regression analysis of biomarkers and microRNAs for the prediction of type 2 diabetes (T2D), expressed as odds ratios (ORs) with 95% confidence intervals (CIs).

Variable	*p*-Value	B	OR	OR 95% CI	
				LI	HI
IL-8 ≥ 20.28 (pg/mL)	0.000	1.907	6.733	3.307	13.709
IL-6 ≥ 7.465 (pg/mL)	0.001	1.490	4.436	2.266	8.987
Leptin ≥ 3.605 (ng/mL)	0.001	2.622	13.765	6.474	29.267
miR-146a ≥ 1.605 (fold change)	0.003	1.580	4.857	1.714	13.767

B: unstandardized regression coefficient for each independent variable in the model, OR: Odds Ratio, LI: low interval, HI: high interval.

**Table 4:** Multivariate logistic regression analysis for Biomarkers and microRNAs to determine a patient’s risk of diabetes and kidney disease.

Variable	*p*-Value	B	OR	OR 95% CI	
				LI	HI
Adiponectin ≥ 8.30 (ug/mL) NC-T2D vs. DKD	0.001	1.424	4.154	1.776	9.718

B: unstandardized regression coefficient for each independent variable in the model, OR: Odds Ratio, LI: low interval, HI: high interval.

## Data Availability

The data presented in this study are available on request from the corresponding author.
